# Supplementary material for: Parental effects of Bt toxin and vitamin A on Helicoverpa armigera
Source: PLoS One. 2022 Jul 6;17(7):e0269585. doi: 10.1371/journal.pone.0269585 (PMC9258806; doi:10.1371/journal.pone.0269585)
Supplement: S1 File — (PDF) [file pone.0269585.s001.pdf]

F1 development data analysis

Diet	Bt toxin	β-Carotene	SEX	Weight	Larval duration
Bt	Bt	No	F	0,2822	30
Bt	Bt	No	F	0,1911	37
Bt	Bt	No	F	0,2424	30
Bt	Bt	No	F	0,2298	25
Bt	Bt	No	F	0,1946	26
Bt	Bt	No	F	0,2887	30
Bt	Bt	No	F	0,2211	30
Bt	Bt	No	F	0,2454	30
Bt	Bt	No	F	0,2587	30
Bt	Bt	No	F	0,235	30
Bt	Bt	No	F	0,1851	34
Bt	Bt	No	F	0,2397	30
Bt	Bt	No	F	0,1142	42
Bt	Bt	No	F	0,243	28
Bt	Bt	No	F	0,2525	28
Bt	Bt	No	M	0,2757	25
Bt	Bt	No	M	0,1999	28
Bt	Bt	No	M	0,1697	34
Bt	Bt	No	M	0,2504	30
Bt	Bt	No	M	0,2372	26
Bt	Bt	No	M	0,2362	28
Bt	Bt	No	M	0,2574	30
Bt	Bt	No	M	0,2544	34
Bt	Bt	No	M	0,2977	31
Bt	Bt	No	M	0,2079	35
Bt	Bt	No	M	0,227	30
Bt	Bt	No	M	0,1908	34
Bt	Bt	No	M	0,2159	27
Bt+β	Bt	Yes	F	0,2048	28
Bt+β	Bt	Yes	F	0,149	37
Bt+β	Bt	Yes	F	0,2281	31
Bt+β	Bt	Yes	F	0,19	30
Bt+β	Bt	Yes	F	0,2135	25
Bt+β	Bt	Yes	F	0,1865	26
Bt+β	Bt	Yes	F	0,2081	28
Bt+β	Bt	Yes	F	0,2363	30
Bt+β	Bt	Yes	F	0,2319	31
Bt+β	Bt	Yes	F	0,222	31
Bt+β	Bt	Yes	F	0,1135	38
Bt+β	Bt	Yes	F	0,275	37
Bt+β	Bt	Yes	F	0,2719	24
Bt+β	Bt	Yes	F	0,2847	22
Bt+β	Bt	Yes	F	0,1954	18
Bt+β	Bt	Yes	F	0,2495	24
Bt+β	Bt	Yes	M	0,1533	37
Bt+β	Bt	Yes	M	0,1703	27
Bt+β	Bt	Yes	M	0,2376	25
Bt+β	Bt	Yes	M	0,2372	37

Bt+β	Bt	Yes	M	0,2435	24
Bt+β	Bt	Yes	M	0,2229	31
Bt+β	Bt	Yes	M	0,2079	29
non-Bt	non-Bt	No	F	0,2968	17
non-Bt	non-Bt	No	F	0,2419	16
non-Bt	non-Bt	No	F	0,2553	17
non-Bt	non-Bt	No	F	0,2778	16
non-Bt	non-Bt	No	F	0,261	18
non-Bt	non-Bt	No	F	0,2667	20
non-Bt	non-Bt	No	F	0,2656	28
non-Bt	non-Bt	No	F	0,3081	24
non-Bt	non-Bt	No	F	0,2787	17
non-Bt	non-Bt	No	F	0,3208	16
non-Bt	non-Bt	No	F	0,2092	18
non-Bt	non-Bt	No	F	0,2463	10
non-Bt	non-Bt	No	F	0,2571	16
non-Bt	non-Bt	No	F	0,237	15
non-Bt	non-Bt	No	M	0,2367	19
non-Bt	non-Bt	No	M	0,3227	17
non-Bt	non-Bt	No	M	0,2696	17
non-Bt	non-Bt	No	M	0,2681	23
non-Bt	non-Bt	No	M	0,2365	20
non-Bt	non-Bt	No	M	0,2919	16
non-Bt	non-Bt	No	M	0,2016	13
non-Bt	non-Bt	No	M	0,2777	17
non-Bt	non-Bt	No	M	0,2823	15
non-Bt	non-Bt	No	M	0,2082	21
non-Bt	non-Bt	No	M	0,1932	13
non-Bt	non-Bt	No	M	0,2719	16
non-Bt	non-Bt	No	M	0,237	15
non Bt+β	non-Bt	Yes	F	0,2271	20
non Bt+β	non-Bt	Yes	F	0,2007	19
non Bt+β	non-Bt	Yes	F	0,2292	17
non Bt+β	non-Bt	Yes	F	0,1744	18
non Bt+β	non-Bt	Yes	F	0,2174	21
non Bt+β	non-Bt	Yes	F	0,2915	24
non Bt+β	non-Bt	Yes	F	0,2231	18
non Bt+β	non-Bt	Yes	F	0,2725	21
non Bt+β	non-Bt	Yes	F	0,2331	23
non Bt+β	non-Bt	Yes	F	0,2019	20
non Bt+β	non-Bt	Yes	F	0,2345	17
non Bt+β	non-Bt	Yes	F	0,2897	24
non Bt+β	non-Bt	Yes	F	0,2416	18
non Bt+β	non-Bt	Yes	F	0,2605	23
non Bt+β	non-Bt	Yes	M	0,2314	17
non Bt+β	non-Bt	Yes	M	0,2523	17
non Bt+β	non-Bt	Yes	M	0,265	24
non Bt+β	non-Bt	Yes	M	0,1932	19
non Bt+β	non-Bt	Yes	M	0,1324	18
non Bt+β	non-Bt	Yes	M	0,2727	24

non Bt+β	non-Bt	Yes	M	0,2294	24
non Bt+β	non-Bt	Yes	M	0,2759	20
non Bt+β	non-Bt	Yes	M	0,2433	17
non Bt+β	non-Bt	Yes	M	0,2381	17

Mating F1 data analysis

GENERACIÓ	REP	Diet	Bt	β-Carotene	STATE	N
F1	1	Bt	Bt	No	**Mated**	5
F1	1	Bt	Bt	No	**Mated**	4
F1	1	Bt	Bt	No	**Mated**	3
F1	3	Bt	Bt	No	**Mated**	1
F1	3	Bt	Bt	No	**Mated**	5
F1	3	Bt	Bt	No	**Mated**	4
F1	1	Bt+β	Bt	Yes	**Mated**	4
F1	1	Bt+β	Bt	Yes	**Mated**	4
F1	1	Bt+β	Bt	Yes	**Mated**	4
F1	3	Bt+β	Bt	Yes	**Mated**	4
F1	3	Bt+β	Bt	Yes	**Mated**	1
F1	3	Bt+β	Bt	Yes	**Mated**	4
F1	1	non-Bt	non-Bt	No	**Mated**	0
F1	1	non-Bt	non-Bt	No	**Mated**	0
F1	1	non-Bt	non-Bt	No	**Mated**	0
F1	3	non-Bt	non-Bt	No	**Mated**	4
F1	3	non-Bt	non-Bt	No	**Mated**	4
F1	3	non-Bt	non-Bt	No	**Mated**	3
F1	1	non-Bt+β	non-Bt	Yes	**Mated**	0
F1	1	non-Bt+β	non-Bt	Yes	**Mated**	0
F1	1	non-Bt+β	non-Bt	Yes	**Mated**	0
F1	3	non-Bt+β	non-Bt	Yes	**Mated**	4
F1	3	non-Bt+β	non-Bt	Yes	**Mated**	6
F1	3	non-Bt+β	non-Bt	Yes	**Mated**	5
F1	1	Bt	Bt	No	**No mated**	1
F1	1	Bt	Bt	No	**No mated**	2
F1	1	Bt	Bt	No	**No mated**	3
F1	3	Bt	Bt	No	**No mated**	5
F1	3	Bt	Bt	No	**No mated**	1
F1	3	Bt	Bt	No	**No mated**	2
F1	1	Bt+β	Bt	Yes	**No mated**	2
F1	1	Bt+β	Bt	Yes	**No mated**	2
F1	1	Bt+β	Bt	Yes	**No mated**	2
F1	3	Bt+β	Bt	Yes	**No mated**	2
F1	3	Bt+β	Bt	Yes	**No mated**	5
F1	3	Bt+β	Bt	Yes	**No mated**	2
F1	1	non-Bt	non-Bt	No	**No mated**	6
F1	1	non-Bt	non-Bt	No	**No mated**	6
F1	1	non-Bt	non-Bt	No	**No mated**	6
F1	3	non-Bt	non-Bt	No	**No mated**	2
F1	3	non-Bt	non-Bt	No	**No mated**	2
F1	3	non-Bt	non-Bt	No	**No mated**	3
F1	1	non-Bt+β	non-Bt	Yes	**No mated**	6
F1	1	non-Bt+β	non-Bt	Yes	**No mated**	6
F1	1	non-Bt+β	non-Bt	Yes	**No mated**	6
F1	3	non-Bt+β	non-Bt	Yes	**No mated**	2
F1	3	non-Bt+β	non-Bt	Yes	**No mated**	0
F1	3	non-Bt+β	non-Bt	Yes	**No mated**	1

Fertility data analysis

GENERACIÓ	REP	Diet	Bt	β-Carotene	Mated females	Larvae	% Mating	Larvae/mated female	PARELL/DIES
F1	1	Bt	Bt	No	5	386	83,33	77,20	3,9
F1	1	Bt	Bt	No	4	1107	66,67	276,75	3,2
F1	1	Bt	Bt	No	3	43	50,00	14,33	3,8
F1	3	Bt	Bt	No	1	572	16,67	572,00	4,3
F1	3	Bt	Bt	No	5	3156	83,33	631,20	4,8
F1	3	Bt	Bt	No	4	4752	66,67	1188,00	6,5
F1	1	Bt+β	Bt	Yes	4	324	66,67	81,00	4,0
F1	1	Bt+β	Bt	Yes	4	660	66,67	165,00	4,0
F1	1	Bt+β	Bt	Yes	4	1202	66,67	300,50	4,1
F1	3	Bt+β	Bt	Yes	4	771	66,67	192,75	4,7
F1	3	Bt+β	Bt	Yes	1	621	16,67	621,00	4,2
F1	3	Bt+β	Bt	Yes	4	1034	66,67	258,50	4,1
F1	1	non-Bt	non-Bt	No	0	0	0,00	0,00	3,1
F1	1	non-Bt	non-Bt	No	0	0	0,00	0,00	4,2
F1	1	non-Bt	non-Bt	No	0	0	0,00	0,00	3,2
F1	3	non-Bt	non-Bt	No	4	1350	66,67	337,50	3,9
F1	3	non-Bt	non-Bt	No	4	2606	66,67	651,50	4,2
F1	3	non-Bt	non-Bt	No	3	1606	50,00	535,33	4,4
F1	1	non-Bt+β	non-Bt	Yes	0	0	0,00	0,00	3,9
F1	1	non-Bt+β	non-Bt	Yes	0	0	0,00	0,00	4,2
F1	1	non-Bt+β	non-Bt	Yes	0	0	0,00	0,00	4,7
F1	3	non-Bt+β	non-Bt	Yes	4	1383	66,67	345,75	4,9
F1	3	non-Bt+β	non-Bt	Yes	6	2936	100,00	489,33	4,9
F1	3	non-Bt+β	non-Bt	Yes	5	1429	83,33	285,80	4,3

F2 evelopment data analysis

Parents	Diet	F1 Bt Tox	F1 β-Car	F2 Bt Tox	F2 β-Car	SEX	Weight	Larval duration
Bt	Bt	Bt	No	Bt	No	M	0,2507	29
Bt	Bt	Bt	No	Bt	No	F	0,2936	33
Bt	Bt	Bt	No	Bt	No	M	0,2339	33
Bt	Bt	Bt	No	Bt	No	F	0,2467	25
Bt	Bt	Bt	No	Bt	No	M	0,307	25
Bt	Bt	Bt	No	Bt	No	M	0,2583	26
Bt	Bt	Bt	No	Bt	No	M	0,3694	26
Bt	Bt	Bt	No	Bt	No	M	0,238	32
Bt	Bt	Bt	No	Bt	No	F	0,19	27
Bt	Bt	Bt	No	Bt	No	F	0,3211	27
Bt	Bt	Bt	No	Bt	No	M	0,3026	27
Bt	Bt	Bt	No	Bt	No	M	0,2329	26
Bt	Bt	Bt	No	Bt	No	M	0,2283	28
Bt	Bt	Bt	No	Bt	No	M	0,1935	43
Bt	Bt	Bt	No	Bt	No	F	0,1864	30
Bt	Bt	Bt	No	Bt	No	F	0,1829	30
Bt	Bt	Bt	No	Bt	No	F	0,2039	30
Bt	Bt	Bt	No	Bt	No	F	0,1686	38
Bt	Bt	Bt	No	Bt	No	F	0,1709	39
Bt	Bt	Bt	No	Bt	No	M	0,1408	33
Bt	Bt	Bt	No	Bt	No	F	0,231	32
Bt	Bt	Bt	No	Bt	No	M	0,2	33
Bt	Bt	Bt	No	Bt	No	M	0,1921	41
Bt	Bt	Bt	No	Bt	No	M	0,1926	36
Bt	Bt	Bt	No	Bt	No	F	0,2299	35
Bt	Bt	Bt	No	Bt	No	F	0,1592	40
Bt	Bt	Bt	No	Bt	No	F	0,1413	45
Bt	Bt	Bt	No	Bt	No	F	0,2536	46
Bt	Bt+β	Bt	No	Bt	Yes	F	0,2536	52
Bt	Bt+β	Bt	No	Bt	Yes	M	0,2177	45
Bt	Bt+β	Bt	No	Bt	Yes	M	0,276	44
Bt	Bt+β	Bt	No	Bt	Yes	M	0,2143	47
Bt	Bt+β	Bt	No	Bt	Yes	F	0,2023	52
Bt	Bt+β	Bt	No	Bt	Yes	F	0,2222	56
Bt	Bt+β	Bt	No	Bt	Yes	M	0,2403	20
Bt	Bt+β	Bt	No	Bt	Yes	M	0,293	20
Bt	Bt+β	Bt	No	Bt	Yes	F	0,1746	17
Bt	Bt+β	Bt	No	Bt	Yes	F	0,1768	20
Bt	Bt+β	Bt	No	Bt	Yes	M	0,2163	19
Bt	Bt+β	Bt	No	Bt	Yes	M	0,2164	20
Bt	Bt+β	Bt	No	Bt	Yes	F	0,142	20
Bt	Bt+β	Bt	No	Bt	Yes	M	0,2076	20
Bt	Bt+β	Bt	No	Bt	Yes	M	0,2121	21
Bt	Bt+β	Bt	No	Bt	Yes	M	0,2016	19
Bt	Bt+β	Bt	No	Bt	Yes	M	0,1847	21
Bt	Bt+β	Bt	No	Bt	Yes	F	0,1444	21
Bt	Bt+β	Bt	No	Bt	Yes	M	0,1499	15
Bt	Bt+β	Bt	No	Bt	Yes	M	0,2263	16
Bt	Bt+β	Bt	No	Bt	Yes	F	0,2497	16
Bt	Bt+β	Bt	No	Bt	Yes	F	0,2	16
Bt	Bt+β	Bt	No	Bt	Yes	F	0,1686	15
Bt	non-Bt	Bt	No	non-Bt	No	F	0,2649	16
Bt	non-Bt	Bt	No	non-Bt	No	M	0,2484	22
Bt	non-Bt	Bt	No	non-Bt	No	F	0,2983	22
Bt	non-Bt	Bt	No	non-Bt	No	F	0,3019	22
Bt	non-Bt	Bt	No	non-Bt	No	F	0,2288	24
Bt	non-Bt	Bt	No	non-Bt	No	M	0,2521	24
Bt	non-Bt	Bt	No	non-Bt	No	F	0,2995	24
Bt	non-Bt	Bt	No	non-Bt	No	M	0,3307	24

Bt	non-Bt	Bt	No	non-Bt	No	M	0,3165	29
Bt	non-Bt	Bt	No	non-Bt	No	M	0,2979	29
Bt	non-Bt	Bt	No	non-Bt	No	M	0,3662	29
Bt	non-Bt	Bt	No	non-Bt	No	M	0,3689	29
Bt	non-Bt	Bt	No	non-Bt	No	F	0,3486	29
Bt	non-Bt	Bt	No	non-Bt	No	F	0,3684	29
Bt	non-Bt	Bt	No	non-Bt	No	F	0,3919	29
Bt	non-Bt	Bt	No	non-Bt	No	F	0,3147	29
Bt	non-Bt	Bt	No	non-Bt	No	F	0,3336	33
Bt	non-Bt	Bt	No	non-Bt	No	F	0,3215	33
Bt	non-Bt	Bt	No	non-Bt	No	F	0,3153	16
Bt	non-Bt	Bt	No	non-Bt	No	M	0,3035	16
Bt	non-Bt	Bt	No	non-Bt	No	F	0,3019	16
Bt	non-Bt	Bt	No	non-Bt	No	F	0,3335	16
Bt	non-Bt	Bt	No	non-Bt	No	M	0,2879	16
Bt	non-Bt	Bt	No	non-Bt	No	M	0,3521	15
Bt	non-Bt	Bt	No	non-Bt	No	F	0,337	15
Bt	non-Bt	Bt	No	non-Bt	No	M	0,305	16
Bt	non-Bt	Bt	No	non-Bt	No	F	0,3786	16
Bt	non-Bt	Bt	No	non-Bt	No	F	0,2818	16
Bt	non-Bt	Bt	No	non-Bt	No	M	0,3029	17
Bt	non-Bt	Bt	No	non-Bt	No	M	0,2802	17
Bt	non-Bt	Bt	No	non-Bt	No	M	0,2979	17
Bt	non-Bt	Bt	No	non-Bt	No	F	0,2809	17
Bt	non-Bt	Bt	No	non-Bt	No	F	0,3117	17
Bt	non-Bt	Bt	No	non-Bt	No	F	0,235	19
Bt	non-Bt	Bt	No	non-Bt	No	M	0,2862	19
Bt	non-Bt	Bt	No	non-Bt	No	M	0,3217	19
Bt	non-Bt	Bt	No	non-Bt	No	M	0,2575	19
Bt	non-Bt	Bt	No	non-Bt	No	F	0,2666	19
Bt	non-Bt	Bt	No	non-Bt	No	M	0,167	19
Bt	non-Bt	Bt	No	non-Bt	No	F	0,2403	20
Bt	non-Bt	Bt	No	non-Bt	No	F	0,27	20
Bt	non-Bt+β	Bt	No	non-Bt	Yes	M	0,2113	21
Bt	non-Bt+β	Bt	No	non-Bt	Yes	F	0,2519	21
Bt	non-Bt+β	Bt	No	non-Bt	Yes	F	0,2144	21
Bt	non-Bt+β	Bt	No	non-Bt	Yes	M	0,2915	22
Bt	non-Bt+β	Bt	No	non-Bt	Yes	M	0,2748	22
Bt	non-Bt+β	Bt	No	non-Bt	Yes	M	0,3262	24
Bt	non-Bt+β	Bt	No	non-Bt	Yes	M	0,3306	24
Bt	non-Bt+β	Bt	No	non-Bt	Yes	M	0,3031	24
Bt	non-Bt+β	Bt	No	non-Bt	Yes	F	0,3095	29
Bt	non-Bt+β	Bt	No	non-Bt	Yes	F	0,308	29
Bt	non-Bt+β	Bt	No	non-Bt	Yes	F	0,3183	33
Bt	non-Bt+β	Bt	No	non-Bt	Yes	M	0,3149	33
Bt	non-Bt+β	Bt	No	non-Bt	Yes	M	0,2861	36
Bt	non-Bt+β	Bt	No	non-Bt	Yes	M	0,3263	39
Bt	non-Bt+β	Bt	No	non-Bt	Yes	M	0,3151	49
Bt	non-Bt+β	Bt	No	non-Bt	Yes	M	0,2936	15
Bt	non-Bt+β	Bt	No	non-Bt	Yes	F	0,306	15
Bt	non-Bt+β	Bt	No	non-Bt	Yes	M	0,3882	15
Bt	non-Bt+β	Bt	No	non-Bt	Yes	M	0,2707	15
Bt	non-Bt+β	Bt	No	non-Bt	Yes	M	0,3385	15
Bt	non-Bt+β	Bt	No	non-Bt	Yes	M	0,3781	15
Bt	non-Bt+β	Bt	No	non-Bt	Yes	F	0,3216	15
Bt	non-Bt+β	Bt	No	non-Bt	Yes	M	0,2892	15
Bt	non-Bt+β	Bt	No	non-Bt	Yes	M	0,2717	15
Bt	non-Bt+β	Bt	No	non-Bt	Yes	F	0,3241	15
Bt	non-Bt+β	Bt	No	non-Bt	Yes	F	0,3105	15
Bt	non-Bt+β	Bt	No	non-Bt	Yes	F	0,2247	15
Bt	non-Bt+β	Bt	No	non-Bt	Yes	F	0,2725	15

Bt	non-Bt+β	Bt	No	non-Bt	Yes	F	0,2683	15
Bt	non-Bt+β	Bt	No	non-Bt	Yes	M	0,252	15
Bt	non-Bt+β	Bt	No	non-Bt	Yes	F	0,2562	15
Bt	non-Bt+β	Bt	No	non-Bt	Yes	M	0,3222	15
Bt	non-Bt+β	Bt	No	non-Bt	Yes	M	0,3578	15
Bt	non-Bt+β	Bt	No	non-Bt	Yes	F	0,3231	15
Bt	non-Bt+β	Bt	No	non-Bt	Yes	F	0,2501	15
Bt	non-Bt+β	Bt	No	non-Bt	Yes	F	0,1821	15
Bt	non-Bt+β	Bt	No	non-Bt	Yes		0,1445	15
Bt+β	Bt	Bt	Yes	Bt	No	F	0,2585	15
Bt+β	Bt	Bt	Yes	Bt	No	M	0,2877	15
Bt+β	Bt	Bt	Yes	Bt	No	F	0,2455	17
Bt+β	Bt	Bt	Yes	Bt	No	F	0,2593	17
Bt+β	Bt	Bt	Yes	Bt	No	M	0,2442	17
Bt+β	Bt	Bt	Yes	Bt	No	F	0,2329	17
Bt+β	Bt	Bt	Yes	Bt	No	F	0,2559	17
Bt+β	Bt	Bt	Yes	Bt	No	M	0,2628	17
Bt+β	Bt	Bt	Yes	Bt	No	F	0,3196	17
Bt+β	Bt	Bt	Yes	Bt	No	F	0,226	17
Bt+β	Bt	Bt	Yes	Bt	No	M	0,2248	17
Bt+β	Bt	Bt	Yes	Bt	No	F	0,2378	17
Bt+β	Bt	Bt	Yes	Bt	No	M	0,2332	17
Bt+β	Bt	Bt	Yes	Bt	No	M	0,1848	17
Bt+β	Bt	Bt	Yes	Bt	No	M	0,1811	17
Bt+β	Bt	Bt	Yes	Bt	No	M	0,207	17
Bt+β	Bt	Bt	Yes	Bt	No	M	0,2795	17
Bt+β	Bt	Bt	Yes	Bt	No	M	0,1627	17
Bt+β	Bt	Bt	Yes	Bt	No	M	0,2263	17
Bt+β	Bt	Bt	Yes	Bt	No	M	0,2239	19
Bt+β	Bt	Bt	Yes	Bt	No	M	0,1614	19
Bt+β	Bt	Bt	Yes	Bt	No	F	0,1622	19
Bt+β	Bt	Bt	Yes	Bt	No	F	0,2278	19
Bt+β	Bt+β	Bt	Yes	Bt	Yes	F	0,2224	19
Bt+β	Bt+β	Bt	Yes	Bt	Yes	F	0,2176	19
Bt+β	Bt+β	Bt	Yes	Bt	Yes	F	0,2339	22
Bt+β	Bt+β	Bt	Yes	Bt	Yes	M	0,1934	22
Bt+β	Bt+β	Bt	Yes	Bt	Yes	M	0,2414	23
Bt+β	Bt+β	Bt	Yes	Bt	Yes	F	0,2716	22
Bt+β	Bt+β	Bt	Yes	Bt	Yes	M	0,2354	22
Bt+β	Bt+β	Bt	Yes	Bt	Yes	M	0,1995	23
Bt+β	Bt+β	Bt	Yes	Bt	Yes	M	0,1932	22
Bt+β	Bt+β	Bt	Yes	Bt	Yes	F	0,2614	23
Bt+β	Bt+β	Bt	Yes	Bt	Yes	M	0,2068	22
Bt+β	Bt+β	Bt	Yes	Bt	Yes	M	0,1803	24
Bt+β	Bt+β	Bt	Yes	Bt	Yes	F	0,2327	22
Bt+β	Bt+β	Bt	Yes	Bt	Yes	F	0,2908	24
Bt+β	Bt+β	Bt	Yes	Bt	Yes	F	0,2768	26
Bt+β	Bt+β	Bt	Yes	Bt	Yes	F	0,1944	25
Bt+β	Bt+β	Bt	Yes	Bt	Yes	M	0,1995	25
Bt+β	Bt+β	Bt	Yes	Bt	Yes	M	0,127	18
Bt+β	Bt+β	Bt	Yes	Bt	Yes	M	0,2506	18
Bt+β	Bt+β	Bt	Yes	Bt	Yes	F	0,2055	20
Bt+β	Bt+β	Bt	Yes	Bt	Yes	F	0,2455	20
Bt+β	Bt+β	Bt	Yes	Bt	Yes	M	0,2202	27
Bt+β	Bt+β	Bt	Yes	Bt	Yes	M	0,1767	26
Bt+β	Bt+β	Bt	Yes	Bt	Yes	F	0,3503	28
Bt+β	Bt+β	Bt	Yes	Bt	Yes	F	0,1843	21
Bt+β	Bt+β	Bt	Yes	Bt	Yes	F	0,2798	21
Bt+β	Bt+β	Bt	Yes	Bt	Yes	F	0,2685	32
Bt+β	Bt+β	Bt	Yes	Bt	Yes	M	0,2227	31
Bt+β	Bt+β	Bt	Yes	Bt	Yes	M	0,1721	22

Bt+β	Bt+β	Bt	Yes	Bt	Yes	M	0,1897	30
Bt+β	Bt+β	Bt	Yes	Bt	Yes	M	0,1896	19
Bt+β	Bt+β	Bt	Yes	Bt	Yes	F	0,2244	19
Bt+β	Bt+β	Bt	Yes	Bt	Yes	M	0,1469	20
Bt+β	Bt+β	Bt	Yes	Bt	Yes	M	0,1827	20
Bt+β	Bt+β	Bt	Yes	Bt	Yes	M	0,1934	31
Bt+β	Bt+β	Bt	Yes	Bt	Yes	F	0,1915	21
Bt+β	Bt+β	Bt	Yes	Bt	Yes	F	0,1734	21
Bt+β	Bt+β	Bt	Yes	Bt	Yes	F	0,1156	25
Bt+β	Bt+β	Bt	Yes	Bt	Yes	F	0,1669	21
Bt+β	Bt+β	Bt	Yes	Bt	Yes	F	0,192	21
Bt+β	Bt+β	Bt	Yes	Bt	Yes	M	0,1078	26
Bt+β	Bt+β	Bt	Yes	Bt	Yes	M	0,1974	34
Bt+β	Bt+β	Bt	Yes	Bt	Yes	M	0,1197	32
Bt+β	Bt+β	Bt	Yes	Bt	Yes	F	0,1516	31
Bt+β	Bt+β	Bt	Yes	Bt	Yes	F	0,0995	45
Bt+β	Bt+β	Bt	Yes	Bt	Yes	F	0,1043	50
Bt+β	Bt+β	Bt	Yes	Bt	Yes	F	0,1369	16
Bt+β	Bt+β	Bt	Yes	Bt	Yes	M	0,1862	17
Bt+β	Bt+β	Bt	Yes	Bt	Yes	M	0,2016	17
Bt+β	non-Bt	Bt	Yes	non-Bt	No	F	0,2649	21
Bt+β	non-Bt	Bt	Yes	non-Bt	No	M	0,2484	21
Bt+β	non-Bt	Bt	Yes	non-Bt	No	M	0,2983	21
Bt+β	non-Bt	Bt	Yes	non-Bt	No	F	0,3019	20
Bt+β	non-Bt	Bt	Yes	non-Bt	No	M	0,2288	20
Bt+β	non-Bt	Bt	Yes	non-Bt	No	M	0,2521	21
Bt+β	non-Bt	Bt	Yes	non-Bt	No	F	0,2995	21
Bt+β	non-Bt	Bt	Yes	non-Bt	No	M	0,3307	21
Bt+β	non-Bt	Bt	Yes	non-Bt	No	M	0,3165	20
Bt+β	non-Bt	Bt	Yes	non-Bt	No	M	0,2979	20
Bt+β	non-Bt	Bt	Yes	non-Bt	No	M	0,3662	21
Bt+β	non-Bt	Bt	Yes	non-Bt	No	M	0,3689	21
Bt+β	non-Bt	Bt	Yes	non-Bt	No	M	0,3486	20
Bt+β	non-Bt	Bt	Yes	non-Bt	No	M	0,3684	22
Bt+β	non-Bt	Bt	Yes	non-Bt	No	M	0,3919	22
Bt+β	non-Bt	Bt	Yes	non-Bt	No	F	0,3147	22
Bt+β	non-Bt	Bt	Yes	non-Bt	No	F	0,3336	21
Bt+β	non-Bt	Bt	Yes	non-Bt	No	M	0,3215	21
Bt+β	non-Bt	Bt	Yes	non-Bt	No	M	0,3153	22
Bt+β	non-Bt	Bt	Yes	non-Bt	No	M	0,3035	22
Bt+β	non-Bt	Bt	Yes	non-Bt	No	M	0,3019	23
Bt+β	non-Bt	Bt	Yes	non-Bt	No	M	0,3335	23
Bt+β	non-Bt	Bt	Yes	non-Bt	No	M	0,2879	22
Bt+β	non-Bt	Bt	Yes	non-Bt	No	F	0,3206	24
Bt+β	non-Bt	Bt	Yes	non-Bt	No	F	0,291	25
Bt+β	non-Bt	Bt	Yes	non-Bt	No	F	0,3453	25
Bt+β	non-Bt	Bt	Yes	non-Bt	No	F	0,2685	23
Bt+β	non-Bt	Bt	Yes	non-Bt	No	F	0,3422	31
Bt+β	non-Bt	Bt	Yes	non-Bt	No	M	0,3178	31
Bt+β	non-Bt	Bt	Yes	non-Bt	No	M	0,3337	27
Bt+β	non-Bt	Bt	Yes	non-Bt	No	M	0,3188	20
Bt+β	non-Bt	Bt	Yes	non-Bt	No	M	0,3034	26
Bt+β	non-Bt	Bt	Yes	non-Bt	No	M	0,3628	19
Bt+β	non-Bt	Bt	Yes	non-Bt	No	M	0,2835	20
Bt+β	non-Bt	Bt	Yes	non-Bt	No	F	0,2851	26
Bt+β	non-Bt	Bt	Yes	non-Bt	No	F	0,341	34
Bt+β	non-Bt	Bt	Yes	non-Bt	No	F	0,2883	32
Bt+β	non-Bt	Bt	Yes	non-Bt	No	F	0,3379	35
Bt+β	non-Bt	Bt	Yes	non-Bt	No	F	0,3846	48
Bt+β	non-Bt	Bt	Yes	non-Bt	No	F	0,3057	17
Bt+β	non-Bt	Bt	Yes	non-Bt	No	M	0,3121	18

Bt+β	non-Bt	Bt	Yes	non-Bt	No	M	0,331	18
Bt+β	non-Bt	Bt	Yes	non-Bt	No	M	0,3136	18
Bt+β	non-Bt	Bt	Yes	non-Bt	No	F	0,2673	18
Bt+β	non-Bt	Bt	Yes	non-Bt	No	F	0,2918	16
Bt+β	non-Bt	Bt	Yes	non-Bt	No	F	0,3054	19
Bt+β	non-Bt	Bt	Yes	non-Bt	No	F	0,3257	19
Bt+β	non-Bt	Bt	Yes	non-Bt	No	F	0,4006	20
Bt+β	non-Bt	Bt	Yes	non-Bt	No	F	0,3357	20
Bt+β	non-Bt	Bt	Yes	non-Bt	No	F	0,3246	18
Bt+β	non-Bt	Bt	Yes	non-Bt	No	F	0,332	20
Bt+β	non-Bt	Bt	Yes	non-Bt	No	F	0,3226	20
Bt+β	non-Bt	Bt	Yes	non-Bt	No	F	0,2403	20
Bt+β	non-Bt	Bt	Yes	non-Bt	No	F	0,279	20
Bt+β	non-Bt+β	Bt	Yes	non-Bt	Yes	M	0,1684	18
Bt+β	non-Bt+β	Bt	Yes	non-Bt	Yes	F	0,2543	20
Bt+β	non-Bt+β	Bt	Yes	non-Bt	Yes	F	0,3064	20
Bt+β	non-Bt+β	Bt	Yes	non-Bt	Yes	M	0,2716	18
Bt+β	non-Bt+β	Bt	Yes	non-Bt	Yes	F	0,3137	21
Bt+β	non-Bt+β	Bt	Yes	non-Bt	Yes	F	0,2637	19
Bt+β	non-Bt+β	Bt	Yes	non-Bt	Yes	F	0,3043	19
Bt+β	non-Bt+β	Bt	Yes	non-Bt	Yes	F	0,3315	20
Bt+β	non-Bt+β	Bt	Yes	non-Bt	Yes	F	0,2852	23
Bt+β	non-Bt+β	Bt	Yes	non-Bt	Yes	M	0,3442	21
Bt+β	non-Bt+β	Bt	Yes	non-Bt	Yes	F	0,2928	24
Bt+β	non-Bt+β	Bt	Yes	non-Bt	Yes	F	0,2912	24
Bt+β	non-Bt+β	Bt	Yes	non-Bt	Yes	M	0,3675	24
Bt+β	non-Bt+β	Bt	Yes	non-Bt	Yes	M	0,3068	25
Bt+β	non-Bt+β	Bt	Yes	non-Bt	Yes	M	0,3348	25
Bt+β	non-Bt+β	Bt	Yes	non-Bt	Yes	M	0,2898	18
Bt+β	non-Bt+β	Bt	Yes	non-Bt	Yes	M	0,2792	18
Bt+β	non-Bt+β	Bt	Yes	non-Bt	Yes	M	0,2716	18
Bt+β	non-Bt+β	Bt	Yes	non-Bt	Yes	F	0,2614	18
Bt+β	non-Bt+β	Bt	Yes	non-Bt	Yes	F	0,331	19
Bt+β	non-Bt+β	Bt	Yes	non-Bt	Yes	F	0,2547	19
Bt+β	non-Bt+β	Bt	Yes	non-Bt	Yes	F	0,3127	21
Bt+β	non-Bt+β	Bt	Yes	non-Bt	Yes	F	0,3209	21
Bt+β	non-Bt+β	Bt	Yes	non-Bt	Yes	F	0,3091	32
Bt+β	non-Bt+β	Bt	Yes	non-Bt	Yes	M	0,3026	32
Bt+β	non-Bt+β	Bt	Yes	non-Bt	Yes	M	0,3182	44
Bt+β	non-Bt+β	Bt	Yes	non-Bt	Yes	M	0,2683	17
Bt+β	non-Bt+β	Bt	Yes	non-Bt	Yes	M	0,3288	17
Bt+β	non-Bt+β	Bt	Yes	non-Bt	Yes	M	0,3234	17
Bt+β	non-Bt+β	Bt	Yes	non-Bt	Yes	F	0,3513	17
Bt+β	non-Bt+β	Bt	Yes	non-Bt	Yes	F	0,3677	18
Bt+β	non-Bt+β	Bt	Yes	non-Bt	Yes	F	0,2455	18
Bt+β	non-Bt+β	Bt	Yes	non-Bt	Yes	M	0,3334	18
Bt+β	non-Bt+β	Bt	Yes	non-Bt	Yes	F	0,296	18
Bt+β	non-Bt+β	Bt	Yes	non-Bt	Yes	M	0,2115	18
Bt+β	non-Bt+β	Bt	Yes	non-Bt	Yes	M	0,4017	17
Bt+β	non-Bt+β	Bt	Yes	non-Bt	Yes	F	0,3974	17
Bt+β	non-Bt+β	Bt	Yes	non-Bt	Yes	M	0,337	19
Bt+β	non-Bt+β	Bt	Yes	non-Bt	Yes	F	0,2941	18
Bt+β	non-Bt+β	Bt	Yes	non-Bt	Yes	F	0,2574	17
Bt+β	non-Bt+β	Bt	Yes	non-Bt	Yes	M	0,3411	16
Bt+β	non-Bt+β	Bt	Yes	non-Bt	Yes	F	0,3347	15
Bt+β	non-Bt+β	Bt	Yes	non-Bt	Yes	M	0,2555	18
Bt+β	non-Bt+β	Bt	Yes	non-Bt	Yes	F	0,2766	19
Bt+β	non-Bt+β	Bt	Yes	non-Bt	Yes	F	0,3229	20
Bt+β	non-Bt+β	Bt	Yes	non-Bt	Yes	F	0,3037	18
Bt+β	non-Bt+β	Bt	Yes	non-Bt	Yes	F	0,2023	20
non-Bt	Bt	non-Bt	No	Bt	No	F	0,2592	19

non-Bt	Bt	non-Bt	No	Bt	No	M	0,2021	20
non-Bt	Bt	non-Bt	No	Bt	No	M	0,2119	20
non-Bt	Bt	non-Bt	No	Bt	No		0,3195	18
non-Bt	Bt	non-Bt	No	Bt	No	M	0,2734	20
non-Bt	Bt	non-Bt	No	Bt	No	M	0,2772	18
non-Bt	Bt	non-Bt	No	Bt	No	M	0,2549	20
non-Bt	Bt	non-Bt	No	Bt	No	M	0,237	20
non-Bt	Bt	non-Bt	No	Bt	No	M	0,251	20
non-Bt	Bt	non-Bt	No	Bt	No	F	0,2676	20
non-Bt	Bt	non-Bt	No	Bt	No	M	0,2052	21
non-Bt	Bt	non-Bt	No	Bt	No	M	0,2014	21
non-Bt	Bt	non-Bt	No	Bt	No	M	0,2636	19
non-Bt	Bt	non-Bt	No	Bt	No	F	0,259	21
non-Bt	Bt	non-Bt	No	Bt	No	F	0,2016	22
non-Bt	Bt	non-Bt	No	Bt	No	M	0,1475	22
non-Bt	Bt	non-Bt	No	Bt	No	F	0,2626	22
non-Bt	Bt	non-Bt	No	Bt	No	M	0,2234	22
non-Bt	Bt	non-Bt	No	Bt	No	M	0,2414	22
non-Bt	Bt	non-Bt	No	Bt	No	M	0,253	22
non-Bt	Bt	non-Bt	No	Bt	No	F	0,2522	22
non-Bt	Bt	non-Bt	No	Bt	No	F	0,203	23
non-Bt	Bt	non-Bt	No	Bt	No	F	0,1343	22
non-Bt	Bt	non-Bt	No	Bt	No	M	0,2511	22
non-Bt	Bt	non-Bt	No	Bt	No	M	0,1736	23
non-Bt	Bt	non-Bt	No	Bt	No	M	0,2346	23
non-Bt	Bt	non-Bt	No	Bt	No	M	0,1861	22
non-Bt	Bt	non-Bt	No	Bt	No	F	0,2263	22
non-Bt	Bt	non-Bt	No	Bt	No	M	0,1785	26
non-Bt	Bt	non-Bt	No	Bt	No	M	0,219	26
non-Bt	Bt	non-Bt	No	Bt	No	M	0,1423	20
non-Bt	Bt	non-Bt	No	Bt	No	M	0,1683	31
non-Bt	Bt	non-Bt	No	Bt	No	M	0,2037	36
non-Bt	Bt	non-Bt	No	Bt	No	F	0,1815	15
non-Bt	Bt	non-Bt	No	Bt	No	F	0,1292	16
non-Bt	Bt	non-Bt	No	Bt	No	F	0,2134	16
non-Bt	Bt	non-Bt	No	Bt	No	F	0,2226	16
non-Bt	Bt	non-Bt	No	Bt	No	F	0,1928	15
non-Bt	Bt	non-Bt	No	Bt	No	F	0,246	15
non-Bt	Bt	non-Bt	No	Bt	No	M	0,1495	16
non-Bt	Bt	non-Bt	No	Bt	No	M	0,133	17
non-Bt	Bt	non-Bt	No	Bt	No	M	0,1461	17
non-Bt	Bt	non-Bt	No	Bt	No	F	0,1111	19
non-Bt	Bt	non-Bt	No	Bt	No	F	0,1234	19
non-Bt	Bt	non-Bt	No	Bt	No	F	0,1413	19
non-Bt	Bt	non-Bt	No	Bt	No	M	0,1957	20
non-Bt	Bt	non-Bt	No	Bt	No	M	0,1727	20
non-Bt	Bt	non-Bt	No	Bt	No	M	0,1621	20
non-Bt	Bt	non-Bt	No	Bt	No	M	0,1771	20
non-Bt	Bt	non-Bt	No	Bt	No	F	0,1075	20
non-Bt	Bt+β	non-Bt	No	Bt	Yes	M	0,2855	20
non-Bt	Bt+β	non-Bt	No	Bt	Yes	F	0,3285	20
non-Bt	Bt+β	non-Bt	No	Bt	Yes	F	0,2796	20
non-Bt	Bt+β	non-Bt	No	Bt	Yes	M	0,305	20
non-Bt	Bt+β	non-Bt	No	Bt	Yes	M	0,2627	20
non-Bt	Bt+β	non-Bt	No	Bt	Yes		0,3057	20
non-Bt	Bt+β	non-Bt	No	Bt	Yes		0,2713	20
non-Bt	Bt+β	non-Bt	No	Bt	Yes		0,2777	20
non-Bt	Bt+β	non-Bt	No	Bt	Yes	M	0,1825	20
non-Bt	Bt+β	non-Bt	No	Bt	Yes	F	0,2752	20
non-Bt	Bt+β	non-Bt	No	Bt	Yes	F	0,2783	21
non-Bt	Bt+β	non-Bt	No	Bt	Yes	M	0,2685	21

non-Bt	Bt+β	non-Bt	No	Bt	Yes	F	0,2442	21
non-Bt	Bt+β	non-Bt	No	Bt	Yes	M	0,2368	21
non-Bt	Bt+β	non-Bt	No	Bt	Yes	M	0,2572	22
non-Bt	Bt+β	non-Bt	No	Bt	Yes	M	0,1904	22
non-Bt	Bt+β	non-Bt	No	Bt	Yes	F	0,2252	22
non-Bt	Bt+β	non-Bt	No	Bt	Yes	M	0,2059	22
non-Bt	Bt+β	non-Bt	No	Bt	Yes	F	0,2109	24
non-Bt	Bt+β	non-Bt	No	Bt	Yes	F	0,2052	24
non-Bt	Bt+β	non-Bt	No	Bt	Yes	F	0,225	24
non-Bt	Bt+β	non-Bt	No	Bt	Yes	M	0,2491	24
non-Bt	Bt+β	non-Bt	No	Bt	Yes	F	0,1909	24
non-Bt	Bt+β	non-Bt	No	Bt	Yes	M	0,1842	24
non-Bt	Bt+β	non-Bt	No	Bt	Yes	M	0,157	29
non-Bt	Bt+β	non-Bt	No	Bt	Yes	F	0,2689	29
non-Bt	Bt+β	non-Bt	No	Bt	Yes		0,1828	29
non-Bt	Bt+β	non-Bt	No	Bt	Yes	M	0,1947	31
non-Bt	Bt+β	non-Bt	No	Bt	Yes	M	0,1608	36
non-Bt	Bt+β	non-Bt	No	Bt	Yes	M	0,1721	16
non-Bt	Bt+β	non-Bt	No	Bt	Yes	F	0,1415	16
non-Bt	Bt+β	non-Bt	No	Bt	Yes	M	0,1773	15
non-Bt	Bt+β	non-Bt	No	Bt	Yes	F	0,1595	17
non-Bt	Bt+β	non-Bt	No	Bt	Yes	M	0,1454	18
non-Bt	Bt+β	non-Bt	No	Bt	Yes	M	0,1729	19
non-Bt	Bt+β	non-Bt	No	Bt	Yes	M	0,1696	20
non-Bt	Bt+β	non-Bt	No	Bt	Yes	F	0,187	17
non-Bt	Bt+β	non-Bt	No	Bt	Yes	M	0,1537	17
non-Bt	Bt+β	non-Bt	No	Bt	Yes	M	0,1359	17
non-Bt	Bt+β	non-Bt	No	Bt	Yes	M	0,1638	20
non-Bt	Bt+β	non-Bt	No	Bt	Yes	M	0,2198	17
non-Bt	Bt+β	non-Bt	No	Bt	Yes	F	0,1853	17
non-Bt	Bt+β	non-Bt	No	Bt	Yes	M	0,1872	17
non-Bt	Bt+β	non-Bt	No	Bt	Yes	F	0,1355	17
non-Bt	Bt+β	non-Bt	No	Bt	Yes	F	0,1601	17
non-Bt	Bt+β	non-Bt	No	Bt	Yes	M	0,1195	17
non-Bt	Bt+β	non-Bt	No	Bt	Yes	M	0,1355	19
non-Bt	Bt+β	non-Bt	No	Bt	Yes	F	0,2206	19
non-Bt	Bt+β	non-Bt	No	Bt	Yes	F	0,1202	19
non-Bt	Bt+β	non-Bt	No	Bt	Yes	F	0,07	19
non-Bt	non-Bt	non-Bt	No	non-Bt	No	F	0,2802	19
non-Bt	non-Bt	non-Bt	No	non-Bt	No	F	0,2799	19
non-Bt	non-Bt	non-Bt	No	non-Bt	No	F	0,3284	19
non-Bt	non-Bt	non-Bt	No	non-Bt	No	M	0,3373	20
non-Bt	non-Bt	non-Bt	No	non-Bt	No	M	0,325	20
non-Bt	non-Bt	non-Bt	No	non-Bt	No	M	0,3487	20
non-Bt	non-Bt	non-Bt	No	non-Bt	No	M	0,2555	20
non-Bt	non-Bt	non-Bt	No	non-Bt	No	M	0,3445	21
non-Bt	non-Bt	non-Bt	No	non-Bt	No	F	0,322	21
non-Bt	non-Bt	non-Bt	No	non-Bt	No	M	0,3424	22
non-Bt	non-Bt	non-Bt	No	non-Bt	No	M	0,357	22
non-Bt	non-Bt	non-Bt	No	non-Bt	No	M	0,2871	24
non-Bt	non-Bt	non-Bt	No	non-Bt	No	M	0,2518	24
non-Bt	non-Bt	non-Bt	No	non-Bt	No	M	0,2683	29
non-Bt	non-Bt	non-Bt	No	non-Bt	No	M	0,2543	29
non-Bt	non-Bt	non-Bt	No	non-Bt	No	M	0,344	29
non-Bt	non-Bt	non-Bt	No	non-Bt	No	F	0,3215	29
non-Bt	non-Bt	non-Bt	No	non-Bt	No	F	0,356	33
non-Bt	non-Bt	non-Bt	No	non-Bt	No	F	0,3068	35
non-Bt	non-Bt	non-Bt	No	non-Bt	No	F	0,3133	15
non-Bt	non-Bt	non-Bt	No	non-Bt	No	M	0,3087	15
non-Bt	non-Bt	non-Bt	No	non-Bt	No	M	0,2678	15
non-Bt	non-Bt	non-Bt	No	non-Bt	No	M	0,3476	15

non-Bt	non-Bt	non-Bt	No	non-Bt	No	M	0,324	15
non-Bt	non-Bt	non-Bt	No	non-Bt	No	M	0,3275	15
non-Bt	non-Bt	non-Bt	No	non-Bt	No	F	0,2819	15
non-Bt	non-Bt	non-Bt	No	non-Bt	No	F	0,2784	15
non-Bt	non-Bt	non-Bt	No	non-Bt	No	F	0,2821	15
non-Bt	non-Bt	non-Bt	No	non-Bt	No	F	0,3239	15
non-Bt	non-Bt	non-Bt	No	non-Bt	No	M	0,3235	15
non-Bt	non-Bt	non-Bt	No	non-Bt	No	F	0,3307	15
non-Bt	non-Bt	non-Bt	No	non-Bt	No	F	0,2493	15
non-Bt	non-Bt	non-Bt	No	non-Bt	No	M	0,32	15
non-Bt	non-Bt	non-Bt	No	non-Bt	No	M	0,319	15
non-Bt	non-Bt	non-Bt	No	non-Bt	No	M	0,3291	15
non-Bt	non-Bt	non-Bt	No	non-Bt	No	M	0,4145	15
non-Bt	non-Bt	non-Bt	No	non-Bt	No	M	0,3363	15
non-Bt	non-Bt	non-Bt	No	non-Bt	No	F	0,287	15
non-Bt	non-Bt	non-Bt	No	non-Bt	No	F	0,3357	15
non-Bt	non-Bt	non-Bt	No	non-Bt	No	M	0,2278	15
non-Bt	non-Bt	non-Bt	No	non-Bt	No	F	0,2667	15
non-Bt	non-Bt	non-Bt	No	non-Bt	No	F	0,1967	15
non-Bt	non-Bt	non-Bt	No	non-Bt	No	M	0,176	15
non-Bt	non-Bt+β	non-Bt	No	non-Bt	Yes	F	0,2875	15
non-Bt	non-Bt+β	non-Bt	No	non-Bt	Yes	M	0,3453	15
non-Bt	non-Bt+β	non-Bt	No	non-Bt	Yes	M	0,2784	15
non-Bt	non-Bt+β	non-Bt	No	non-Bt	Yes	M	0,279	15
non-Bt	non-Bt+β	non-Bt	No	non-Bt	Yes	F	0,3437	15
non-Bt	non-Bt+β	non-Bt	No	non-Bt	Yes	M	0,324	15
non-Bt	non-Bt+β	non-Bt	No	non-Bt	Yes	M	0,2986	15
non-Bt	non-Bt+β	non-Bt	No	non-Bt	Yes	F	0,2781	15
non-Bt	non-Bt+β	non-Bt	No	non-Bt	Yes	M	0,3313	17
non-Bt	non-Bt+β	non-Bt	No	non-Bt	Yes	F	0,2634	17
non-Bt	non-Bt+β	non-Bt	No	non-Bt	Yes	F	0,3002	17
non-Bt	non-Bt+β	non-Bt	No	non-Bt	Yes	F	0,3095	17
non-Bt	non-Bt+β	non-Bt	No	non-Bt	Yes	F	0,3087	17
non-Bt	non-Bt+β	non-Bt	No	non-Bt	Yes	F	0,2907	17
non-Bt	non-Bt+β	non-Bt	No	non-Bt	Yes	M	0,2869	17
non-Bt	non-Bt+β	non-Bt	No	non-Bt	Yes	F	0,2286	17
non-Bt	non-Bt+β	non-Bt	No	non-Bt	Yes	F	0,293	17
non-Bt	non-Bt+β	non-Bt	No	non-Bt	Yes	M	0,3179	17
non-Bt	non-Bt+β	non-Bt	No	non-Bt	Yes	F	0,2955	17
non-Bt	non-Bt+β	non-Bt	No	non-Bt	Yes	F	0,2962	19
non-Bt	non-Bt+β	non-Bt	No	non-Bt	Yes	M	0,3214	19
non-Bt	non-Bt+β	non-Bt	No	non-Bt	Yes	M	0,3139	22
non-Bt	non-Bt+β	non-Bt	No	non-Bt	Yes	M	0,328	22
non-Bt	non-Bt+β	non-Bt	No	non-Bt	Yes	F	0,3414	20
non-Bt	non-Bt+β	non-Bt	No	non-Bt	Yes	F	0,317	24
non-Bt	non-Bt+β	non-Bt	No	non-Bt	Yes	F	0,2895	21
non-Bt	non-Bt+β	non-Bt	No	non-Bt	Yes	F	0,2707	23
non-Bt	non-Bt+β	non-Bt	No	non-Bt	Yes	M	0,2377	21
non-Bt	non-Bt+β	non-Bt	No	non-Bt	Yes	F	0,3057	21
non-Bt	non-Bt+β	non-Bt	No	non-Bt	Yes	M	0,2872	22
non-Bt	non-Bt+β	non-Bt	No	non-Bt	Yes			22
non-Bt	non-Bt+β	non-Bt	No	non-Bt	Yes	F	0,3227	23
non-Bt	non-Bt+β	non-Bt	No	non-Bt	Yes	F	0,303	21
non-Bt	non-Bt+β	non-Bt	No	non-Bt	Yes	M	0,3015	24
non-Bt	non-Bt+β	non-Bt	No	non-Bt	Yes	F	0,2894	23
non-Bt	non-Bt+β	non-Bt	No	non-Bt	Yes	F	0,3708	23
non-Bt	non-Bt+β	non-Bt	No	non-Bt	Yes	M	0,3117	23
non-Bt	non-Bt+β	non-Bt	No	non-Bt	Yes	M	0,2233	25
non-Bt	non-Bt+β	non-Bt	No	non-Bt	Yes			18
non-Bt	non-Bt+β	non-Bt	No	non-Bt	Yes	M	0,2715	17
non-Bt	non-Bt+β	non-Bt	No	non-Bt	Yes	F	0,3355	29

non-Bt	non-Bt+β	non-Bt	No	non-Bt	Yes			21
non-Bt	non-Bt+β	non-Bt	No	non-Bt	Yes	M	0,2456	21
non-Bt	non-Bt+β	non-Bt	No	non-Bt	Yes	M	0,2639	22
non-Bt	non-Bt+β	non-Bt	No	non-Bt	Yes			22
non-Bt+β	Bt	non-Bt	Yes	Bt	No	M	0,2976	22
non-Bt+β	Bt	non-Bt	Yes	Bt	No		0,3254	18
non-Bt+β	Bt	non-Bt	Yes	Bt	No	F	0,173	18
non-Bt+β	Bt	non-Bt	Yes	Bt	No	M	0,2442	20
non-Bt+β	Bt	non-Bt	Yes	Bt	No	F	0,201	20
non-Bt+β	Bt	non-Bt	Yes	Bt	No	M	0,3586	20
non-Bt+β	Bt	non-Bt	Yes	Bt	No	M	0,2123	20
non-Bt+β	Bt	non-Bt	Yes	Bt	No	M	0,1934	20
non-Bt+β	Bt	non-Bt	Yes	Bt	No	M	0,2243	
non-Bt+β	Bt	non-Bt	Yes	Bt	No	M	0,2113	21
non-Bt+β	Bt	non-Bt	Yes	Bt	No	F	0,2052	21
non-Bt+β	Bt	non-Bt	Yes	Bt	No	F	0,1912	21
non-Bt+β	Bt	non-Bt	Yes	Bt	No	M	0,2733	22
non-Bt+β	Bt	non-Bt	Yes	Bt	No	F	0,2879	22
non-Bt+β	Bt	non-Bt	Yes	Bt	No	M	0,1433	22
non-Bt+β	Bt	non-Bt	Yes	Bt	No	F	0,206	24
non-Bt+β	Bt	non-Bt	Yes	Bt	No	F	0,2317	35
non-Bt+β	Bt	non-Bt	Yes	Bt	No	M	0,2589	24
non-Bt+β	Bt	non-Bt	Yes	Bt	No	M	0,2497	24
non-Bt+β	Bt	non-Bt	Yes	Bt	No	F	0,2603	24
non-Bt+β	Bt	non-Bt	Yes	Bt	No	F	0,2157	25
non-Bt+β	Bt	non-Bt	Yes	Bt	No		0,16	28
non-Bt+β	Bt	non-Bt	Yes	Bt	No		0,1427	42
non-Bt+β	Bt	non-Bt	Yes	Bt	No		0,2184	16
non-Bt+β	Bt	non-Bt	Yes	Bt	No	F	0,2601	16
non-Bt+β	Bt	non-Bt	Yes	Bt	No	F	0,1225	17
non-Bt+β	Bt	non-Bt	Yes	Bt	No	F	0,2719	17
non-Bt+β	Bt	non-Bt	Yes	Bt	No	M	0,2447	17
non-Bt+β	Bt	non-Bt	Yes	Bt	No	M	0,2476	18
non-Bt+β	Bt	non-Bt	Yes	Bt	No	M	0,2188	17
non-Bt+β	Bt	non-Bt	Yes	Bt	No	F	0,2027	20
non-Bt+β	Bt	non-Bt	Yes	Bt	No	F	0,1254	21
non-Bt+β	Bt	non-Bt	Yes	Bt	No	F	0,165	21
non-Bt+β	Bt	non-Bt	Yes	Bt	No	F	0,1225	20
non-Bt+β	Bt	non-Bt	Yes	Bt	No	M	0,1974	20
non-Bt+β	Bt	non-Bt	Yes	Bt	No	F	0,1188	21
non-Bt+β	Bt	non-Bt	Yes	Bt	No	F	0,1863	20
non-Bt+β	Bt	non-Bt	Yes	Bt	No	M	0,1781	21
non-Bt+β	Bt	non-Bt	Yes	Bt	No	M	0,1923	21
non-Bt+β	Bt	non-Bt	Yes	Bt	No	F	0,175	21
non-Bt+β	Bt+β	non-Bt	Yes	Bt	Yes	M	0,2816	21
non-Bt+β	Bt+β	non-Bt	Yes	Bt	Yes	F	0,3133	21
non-Bt+β	Bt+β	non-Bt	Yes	Bt	Yes	M	0,2947	22
non-Bt+β	Bt+β	non-Bt	Yes	Bt	Yes	F	0,1709	22
non-Bt+β	Bt+β	non-Bt	Yes	Bt	Yes	F	0,2318	23
non-Bt+β	Bt+β	non-Bt	Yes	Bt	Yes	F	0,213	23
non-Bt+β	Bt+β	non-Bt	Yes	Bt	Yes	F	0,2289	24
non-Bt+β	Bt+β	non-Bt	Yes	Bt	Yes	F	0,2071	18
non-Bt+β	Bt+β	non-Bt	Yes	Bt	Yes	F	0,2756	24
non-Bt+β	Bt+β	non-Bt	Yes	Bt	Yes	F	0,251	24
non-Bt+β	Bt+β	non-Bt	Yes	Bt	Yes	F	0,2814	28
non-Bt+β	Bt+β	non-Bt	Yes	Bt	Yes	F	0,201	26
non-Bt+β	Bt+β	non-Bt	Yes	Bt	Yes	F	0,1308	26
non-Bt+β	Bt+β	non-Bt	Yes	Bt	Yes	F	0,2305	26
non-Bt+β	Bt+β	non-Bt	Yes	Bt	Yes	F	0,1708	31
non-Bt+β	Bt+β	non-Bt	Yes	Bt	Yes	F	0,2151	22
non-Bt+β	Bt+β	non-Bt	Yes	Bt	Yes	F		28

non-Bt+β	Bt+β	non-Bt	Yes	Bt	Yes	F	0,207	26
non-Bt+β	Bt+β	non-Bt	Yes	Bt	Yes	F	0,154	32
non-Bt+β	Bt+β	non-Bt	Yes	Bt	Yes	F	0,1466	45
non-Bt+β	Bt+β	non-Bt	Yes	Bt	Yes	F	0,1647	
non-Bt+β	Bt+β	non-Bt	Yes	Bt	Yes		0,1755	17
non-Bt+β	Bt+β	non-Bt	Yes	Bt	Yes		0,125	17
non-Bt+β	Bt+β	non-Bt	Yes	Bt	Yes	F	0,163	17
non-Bt+β	Bt+β	non-Bt	Yes	Bt	Yes	F	0,1727	16
non-Bt+β	non-Bt	non-Bt	Yes	non-Bt	No	F	0,2713	19
non-Bt+β	non-Bt	non-Bt	Yes	non-Bt	No	M	0,2803	17
non-Bt+β	non-Bt	non-Bt	Yes	non-Bt	No	F	0,2592	19
non-Bt+β	non-Bt	non-Bt	Yes	non-Bt	No	M	0,2892	19
non-Bt+β	non-Bt	non-Bt	Yes	non-Bt	No	M	0,3144	19
non-Bt+β	non-Bt	non-Bt	Yes	non-Bt	No	F	0,3129	19
non-Bt+β	non-Bt	non-Bt	Yes	non-Bt	No	M	0,3323	17
non-Bt+β	non-Bt	non-Bt	Yes	non-Bt	No	F	0,3463	20
non-Bt+β	non-Bt	non-Bt	Yes	non-Bt	No	M	0,2993	20
non-Bt+β	non-Bt	non-Bt	Yes	non-Bt	No	F	0,2541	18
non-Bt+β	non-Bt	non-Bt	Yes	non-Bt	No	F	0,3148	18
non-Bt+β	non-Bt	non-Bt	Yes	non-Bt	No	M	0,3156	18
non-Bt+β	non-Bt	non-Bt	Yes	non-Bt	No	F	0,2809	20
non-Bt+β	non-Bt	non-Bt	Yes	non-Bt	No	M	0,3273	21
non-Bt+β	non-Bt	non-Bt	Yes	non-Bt	No	F	0,3177	21
non-Bt+β	non-Bt	non-Bt	Yes	non-Bt	No	M	0,3323	19
non-Bt+β	non-Bt	non-Bt	Yes	non-Bt	No	F	0,3178	19
non-Bt+β	non-Bt	non-Bt	Yes	non-Bt	No	F	0,333	21
non-Bt+β	non-Bt	non-Bt	Yes	non-Bt	No	F	0,37	22
non-Bt+β	non-Bt	non-Bt	Yes	non-Bt	No	F	0,2762	21
non-Bt+β	non-Bt	non-Bt	Yes	non-Bt	No	F	0,3358	21
non-Bt+β	non-Bt	non-Bt	Yes	non-Bt	No	M	0,3153	23
non-Bt+β	non-Bt	non-Bt	Yes	non-Bt	No	M	0,3313	16
non-Bt+β	non-Bt	non-Bt	Yes	non-Bt	No	F	0,3563	25
non-Bt+β	non-Bt	non-Bt	Yes	non-Bt	No	F	0,3639	26
non-Bt+β	non-Bt	non-Bt	Yes	non-Bt	No	M	0,3344	19
non-Bt+β	non-Bt	non-Bt	Yes	non-Bt	No	F	0,329	19
non-Bt+β	non-Bt	non-Bt	Yes	non-Bt	No	F	0,3113	28
non-Bt+β	non-Bt	non-Bt	Yes	non-Bt	No	M	0,3815	20
non-Bt+β	non-Bt	non-Bt	Yes	non-Bt	No	M	0,3291	29
non-Bt+β	non-Bt	non-Bt	Yes	non-Bt	No	F	0,3081	21
non-Bt+β	non-Bt	non-Bt	Yes	non-Bt	No	F	0,2958	31
non-Bt+β	non-Bt	non-Bt	Yes	non-Bt	No	M	0,2682	31
non-Bt+β	non-Bt	non-Bt	Yes	non-Bt	No	M	0,3181	17
non-Bt+β	non-Bt	non-Bt	Yes	non-Bt	No	M	0,338	17
non-Bt+β	non-Bt	non-Bt	Yes	non-Bt	No	M	0,288	17
non-Bt+β	non-Bt	non-Bt	Yes	non-Bt	No	F	0,2909	17
non-Bt+β	non-Bt	non-Bt	Yes	non-Bt	No	F	0,3584	18
non-Bt+β	non-Bt	non-Bt	Yes	non-Bt	No	M	0,2702	18
non-Bt+β	non-Bt	non-Bt	Yes	non-Bt	No	F	0,2237	18
non-Bt+β	non-Bt	non-Bt	Yes	non-Bt	No	F	0,1977	18
non-Bt+β	non-Bt	non-Bt	Yes	non-Bt	No	M	0,2204	17
non-Bt+β	non-Bt+β	non-Bt	Yes	non-Bt	Yes	F	0,3074	19
non-Bt+β	non-Bt+β	non-Bt	Yes	non-Bt	Yes	F	0,3459	19
non-Bt+β	non-Bt+β	non-Bt	Yes	non-Bt	Yes	F	0,2536	19
non-Bt+β	non-Bt+β	non-Bt	Yes	non-Bt	Yes	M	0,2644	19
non-Bt+β	non-Bt+β	non-Bt	Yes	non-Bt	Yes	M	0,3015	20
non-Bt+β	non-Bt+β	non-Bt	Yes	non-Bt	Yes	M	0,3208	19
non-Bt+β	non-Bt+β	non-Bt	Yes	non-Bt	Yes	F	0,3019	20
non-Bt+β	non-Bt+β	non-Bt	Yes	non-Bt	Yes	F	0,3601	20
non-Bt+β	non-Bt+β	non-Bt	Yes	non-Bt	Yes	M	0,3175	20
non-Bt+β	non-Bt+β	non-Bt	Yes	non-Bt	Yes	F	0,3053	20
non-Bt+β	non-Bt+β	non-Bt	Yes	non-Bt	Yes	M	0,2751	20

non-Bt+β	non-Bt+β	non-Bt	Yes	non-Bt	Yes	F	0,2925	19
non-Bt+β	non-Bt+β	non-Bt	Yes	non-Bt	Yes	F	0,3303	20
non-Bt+β	non-Bt+β	non-Bt	Yes	non-Bt	Yes	F	0,3361	19
non-Bt+β	non-Bt+β	non-Bt	Yes	non-Bt	Yes	F	0,3185	20
non-Bt+β	non-Bt+β	non-Bt	Yes	non-Bt	Yes	M	0,2473	21
non-Bt+β	non-Bt+β	non-Bt	Yes	non-Bt	Yes	F	0,2938	21
non-Bt+β	non-Bt+β	non-Bt	Yes	non-Bt	Yes	M	0,359	21
non-Bt+β	non-Bt+β	non-Bt	Yes	non-Bt	Yes	F	0,3633	21
non-Bt+β	non-Bt+β	non-Bt	Yes	non-Bt	Yes	F	0,2929	21
non-Bt+β	non-Bt+β	non-Bt	Yes	non-Bt	Yes	M	0,355	20
non-Bt+β	non-Bt+β	non-Bt	Yes	non-Bt	Yes	F	0,3451	21
non-Bt+β	non-Bt+β	non-Bt	Yes	non-Bt	Yes	M	0,3304	19
non-Bt+β	non-Bt+β	non-Bt	Yes	non-Bt	Yes	M	0,3883	21
non-Bt+β	non-Bt+β	non-Bt	Yes	non-Bt	Yes	F	0,2957	20
non-Bt+β	non-Bt+β	non-Bt	Yes	non-Bt	Yes	M	0,3355	22
non-Bt+β	non-Bt+β	non-Bt	Yes	non-Bt	Yes	F	0,2953	22
non-Bt+β	non-Bt+β	non-Bt	Yes	non-Bt	Yes	F	0,293	23
non-Bt+β	non-Bt+β	non-Bt	Yes	non-Bt	Yes	M	0,2641	24
non-Bt+β	non-Bt+β	non-Bt	Yes	non-Bt	Yes	M	0,2707	25
non-Bt+β	non-Bt+β	non-Bt	Yes	non-Bt	Yes	M	0,3323	25
non-Bt+β	non-Bt+β	non-Bt	Yes	non-Bt	Yes	F	0,2685	18
non-Bt+β	non-Bt+β	non-Bt	Yes	non-Bt	Yes	F	0,2585	18
non-Bt+β	non-Bt+β	non-Bt	Yes	non-Bt	Yes	M	0,2607	27
non-Bt+β	non-Bt+β	non-Bt	Yes	non-Bt	Yes	F	0,2314	20
non-Bt+β	non-Bt+β	non-Bt	Yes	non-Bt	Yes	F	0,3182	30
non-Bt+β	non-Bt+β	non-Bt	Yes	non-Bt	Yes	F	0,1747	26
non-Bt+β	non-Bt+β	non-Bt	Yes	non-Bt	Yes	F	0,1902	34

Mortality data analysis

Generation	Parents	Diet	Bt toxin	β-Carotene	Dead	Pupae	TOTAL	% Mortality
1	field	Bt	Bt	No	2	28	30	7
1	field	Bt+β	Bt	Yes	7	23	30	23
1	field	non-Bt	non-Bt	No	5	27	32	16
1	field	non-Bt+β	non-Bt	Yes	3	24	27	11
2	Bt	Bt	Bt	No	67	28	95	71
2	Bt	Bt+β	Bt	Yes	54	23	77	70
2	Bt	non-Bt	non-Bt	No	19	41	60	32
2	Bt	non-Bt+β	non-Bt	Yes	16	37	53	30
2	Bt+β	Bt	Bt	No	48	23	71	68
2	Bt+β	Bt+β	Bt	Yes	54	49	103	52
2	Bt+β	non-Bt	non-Bt	No	7	54	61	11
2	Bt+β	non-Bt+β	non-Bt	Yes	12	47	59	20
2	non-Bt	Bt	Bt	No	98	50	148	67
2	non-Bt	Bt+β	Bt	Yes	97	50	147	66
2	non-Bt	non-Bt	non-Bt	No	43	43	86	50
2	non-Bt	non-Bt+β	non-Bt	Yes	45	41	86	51
2	non-Bt+β	Bt	Bt	No	97	40	137	71
2	non-Bt+β	Bt+β	Bt	Yes	95	24	119	79
2	non-Bt+β	non-Bt	non-Bt	No	24	42	66	36
2	non-Bt+β	non-Bt+β	non-Bt	Yes	27	38	65	42

Mortality chi analysis data

Generation	Parents	Diet	Bt toxin	β-Carotene	state	N
1	field	Bt	Bt	No	**Dead**	2
1	field	Bt+β	Bt	Yes	**Dead**	7
1	field	non-Bt	non-Bt	No	**Dead**	5
1	field	non-Bt+β	non-Bt	Yes	**Dead**	3
1	field	Bt	Bt	No	**Pupa**	28
1	field	Bt+β	Bt	Yes	**Pupa**	23
1	field	non-Bt	non-Bt	No	**Pupa**	27
1	field	non-Bt+β	non-Bt	Yes	**Pupa**	24
2	Bt	Bt	Bt	No	**Dead**	67
2	Bt	Bt+β	Bt	Yes	**Dead**	54
2	Bt	non-Bt	non-Bt	No	**Dead**	19
2	Bt	non-Bt+β	non-Bt	Yes	**Dead**	16
2	Bt	Bt	Bt	No	**Pupa**	28
2	Bt	Bt+β	Bt	Yes	**Pupa**	23
2	Bt	non-Bt	non-Bt	No	**Pupa**	41
2	Bt	non-Bt+β	non-Bt	Yes	**Pupa**	37
2	Bt+β	Bt	Bt	No	**Dead**	48
2	Bt+β	Bt+β	Bt	Yes	**Dead**	54
2	Bt+β	non-Bt	non-Bt	No	**Dead**	7
2	Bt+β	non-Bt+β	non-Bt	Yes	**Dead**	12
2	Bt+β	Bt	Bt	No	**Pupa**	23
2	Bt+β	Bt+β	Bt	Yes	**Pupa**	49
2	Bt+β	non-Bt	non-Bt	No	**Pupa**	54
2	Bt+β	non-Bt+β	non-Bt	Yes	**Pupa**	47
2	non-Bt	Bt	Bt	No	**Dead**	98
2	non-Bt	Bt+β	Bt	Yes	**Dead**	97
2	non-Bt	non-Bt	non-Bt	No	**Dead**	43
2	non-Bt	non-Bt+β	non-Bt	Yes	**Dead**	45
2	non-Bt	Bt	Bt	No	**Pupa**	50
2	non-Bt	Bt+β	Bt	Yes	**Pupa**	50
2	non-Bt	non-Bt	non-Bt	No	**Pupa**	43
2	non-Bt	non-Bt+β	non-Bt	Yes	**Pupa**	41
2	non-Bt+β	Bt	Bt	No	**Dead**	97
2	non-Bt+β	Bt+β	Bt	Yes	**Dead**	95
2	non-Bt+β	non-Bt	non-Bt	No	**Dead**	24
2	non-Bt+β	non-Bt+β	non-Bt	Yes	**Dead**	27
2	non-Bt+β	Bt	Bt	No	**Pupa**	40
2	non-Bt+β	Bt+β	Bt	Yes	**Pupa**	24
2	non-Bt+β	non-Bt	non-Bt	No	**Pupa**	42
2	non-Bt+β	non-Bt+β	non-Bt	Yes	**Pupa**	38
