# Supplementary material for: Parental effects of Bt toxin and vitamin A on Helicoverpa armigera
Source: PLoS One. 2022 Jul 6;17(7):e0269585. doi: 10.1371/journal.pone.0269585 (PMC9258806; doi:10.1371/journal.pone.0269585)
Supplement: Attachment — Submitted filename: Response to Reviewers.docx [file pone.0269585.s002.docx]

PONE-D-22-04499

Parental effects of Bt toxin and vitamin A on Helicoverpa armigera

PLOS ONE
Patrizia Falabella Academic Editor and Reviewer's Questions
PLOS ONE

Journal Requirements:

When submitting your revision, we need you to address these additional requirements.

1. Please ensure that your manuscript meets PLOS ONE's style requirements, including those for file naming. The PLOS ONE style templates can be found at https://journals.plos.org/plosone/s/file?id=wjVg/PLOSOne_formatting_sample_main_body.pdf and 
https://journals.plos.org/plosone/s/file?id=ba62/PLOSOne_formatting_sample_title_authors_affiliations.pdf.

2. We note that the grant information you provided in the ‘Funding Information’ and ‘Financial Disclosure’ sections do not match.

When you resubmit, please ensure that you provide the correct grant numbers for the awards you received for your study in the ‘Funding Information’ section. Now Corrected

3. Thank you for stating the following in the Acknowledgments Section of your manuscript: 
[We are grateful to Ministerio de Ciencia e Innovación (Spanish government) for financial support. The authors thank Teresa Estela her administrative support and Joan Safont for his invaluable help in the general rearing of insects. Thanks to Professor Ramon Albajes for his unconditional support.]

We note that you have provided funding information that is not currently declared in your Funding Statement. However, funding information should not appear in the Acknowledgments section or other areas of your manuscript. We will only publish funding information present in the Funding Statement section of the online submission form. 
Please remove any funding-related text from the manuscript and let us know how you would like to update your Funding Statement. Currently, your Funding Statement reads as follows: 
 [The funders had no role in study design, data collection and analysis, decision to publish, or preparation of the manuscript.]

Please include your amended statements within your cover letter; we will change the online submission form on your behalf. Done

4. Please include your full ethics statement in the ‘Methods’ section of your manuscript file. In your statement, please include the full name of the IRB or ethics committee who approved or waived your study, as well as whether or not you obtained informed written or verbal consent. If consent was waived for your study, please include this information in your statement as well. Done, Lines 106-108

[Note: HTML markup is below. Please do not edit.]

Reviewers' comments:

Reviewer's Responses to Questions

**Comments to the Author**

1. Is the manuscript technically sound, and do the data support the conclusions?

The manuscript must describe a technically sound piece of scientific research with data that supports the conclusions. Experiments must have been conducted rigorously, with appropriate controls, replication, and sample sizes. The conclusions must be drawn appropriately based on the data presented.

Reviewer #1: Yes

Reviewer #2: Yes

2. Has the statistical analysis been performed appropriately and rigorously?

Reviewer #1: Yes

Reviewer #2: Yes

3. Have the authors made all data underlying the findings in their manuscript fully available?

The [PLOS Data policy](http://www.plosone.org/static/policies.action#sharing) requires authors to make all data underlying the findings described in their manuscript fully available without restriction, with rare exception (please refer to the Data Availability Statement in the manuscript PDF file). The data should be provided as part of the manuscript or its supporting information, or deposited to a public repository. For example, in addition to summary statistics, the data points behind means, medians and variance measures should be available. If there are restrictions on publicly sharing data—e.g. participant privacy or use of data from a third party—those must be specified.

Reviewer #1: Yes

Reviewer #2: Yes

4. Is the manuscript presented in an intelligible fashion and written in standard English?

PLOS ONE does not copyedit accepted manuscripts, so the language in submitted articles must be clear, correct, and unambiguous. Any typographical or grammatical errors should be corrected at revision, so please note any specific errors here.

Reviewer #1: No The document has now been revised by an official English Editing services. We have the certificate showing it.

Reviewer #2: Yes

5. Review Comments to the Author

Please use the space provided to explain your answers to the questions above. You may also include additional comments for the author, including concerns about dual publication, research ethics, or publication ethics. (Please upload your review as an attachment if it exceeds 20,000 characters)

Reviewer #1: Comments:
This paper by López et al. “Parental effects of Bt toxin and vitamin A on Helicoverpa armigera” has researched the effect of vitamin A (β-carotene) and Bt protein on H. armigera larvae and their offspring. The work could be helpful to better understand the transgenerational effect of vitamin and Bt protein, and the potential resistance of H. armigera to Bt crops. In general, the experimental design of the paper is good and the data provided consequent, the information amassed is interesting. However, the manuscript is not acceptable before some experiment should be added. Meanwhile, the language quality of the MS should be improved. I hope my comments could help this interesting work

Introduction

The paragraphs of the Introduction is much too long. And there are too many long sentences, many of them are hard to understand. Done. The document has now been revised by an official English Editing services

Materials and methods
Insect rearing
“H. armigera larvae were collected in maize or alfalfa crops in Lleida”. Host adaptability of insect has been found to be dependent on many factors, the authors need to be clear that the larvae were collected from maize? or alfalfa crops?

The larvae of all experiments were collected only in alfalfa fields. Now this point has been corrected in M&M Line 106

Diets
“……, a 25% of maize flour was replaced by lyophilized Bt or non-Bt maize leaves, adding or not 0.6 % of β-Carotene”. The author needs to explain that why diets and 25% maize folur, 0.6% β-Carotene. Is there any literature on this? What was the dosage of Cry1Ab protein in 25% of Bt maize flour? Done. We have rewritten lines 128-135 adding the data and necessary references

Experiments
Experiments with the F1 larvae and adults
“Larval mortality, duration of larval development and pupal weight were recorded”. Were the insect observed and recorded the mortality daily? Yes, daily, line 148.

What is the standard of death? We consider the standard mortality, the mortality on the non-Bt diet

Why were there 6 females and 5 males in the mating cage? In the M&M, Insect General Rearing we have added the explanation with the reference of why we used 6 females and 5 males per cage, lines 113-115

Please clearly state the number of insect for each experiment. Each experiment indicates the number on insects tested. Lines 144, 170

Statistical analysis
“using a generalized linear model with a binomial distribution” corrected to “using a generalized linear model (GLM) with a binomial distribution”. I recommend that you use this symbol instead of the common abbreviations throughout the manuscript. Done

Results
F1 mating and fertility
“……but neither β-carotene nor the interaction Bt-β-carotene had any effect”, the result of this sentence was not show in Fig. 4. We apologized for the unclear presentation of these results. Now, we have added the statistics of the effect of β-carotene in lines 243-245, we have also modified the X-axis the Figure 4 and the Figure 4 caption (lines 250-252) to explain in a better way.

F2 Development and Mortality
Can the authors clarify why they did not research on the β-carotene to F2 development and mortality? Figure note should be added in Fig. 7. Again, we apologized for the unclear presentation of these results. Now, we have added the statistics of the effect of β-carotene in lines 296-297, we have also modified the X-axis in the Figure 7, and the Figure 7 caption (lines 302-304) to explain in a better way.


Discussion
The paragraphs of the Discussion is much too long

Done. The document has been revised.

“However, the life-history-traits affected by phytophagous feeding……”, please cite literature references. The literature references appear in L 310-312
“……in previous work with the same species [8] and with other nearby species such as H. zea [22]”, the authors should be discussed the differences between references and this research. Done, lines 327-330

“Maternal o paternal effects due to……” corrected to “Maternal or paternal effects due to……” Changed in the new version, line 358

Reviewer #2: Reviewer Comments for PONE-D-22-04499
The manuscript entitled “Parental effects of Bt toxin and vitamin A on Helicoverpa armigera” by
Carmen López, Daniela Zanga, Alejandro Juárez-Escario, Pilar Muñoz, Matilde Eizaguirre provides advances in the existing knowledge on Bt toxins for crop protection.
Introduction
developed instead of development Changed in the new version
Add more reference against each already mentioned “H. armigera is a serious cosmopolitan insect pest whose larvae are polyphagous [9] and have a high capacity to develop resistance to insecticides [10] and to the Bt toxin [11]. Done, added more references. L76-78
Rephrase “that feed on maize mainly on the silks (styles) among many other crops such as cotton or alfalfa.” Eliminated in the new version
(to the Bt toxin) and although part of the larvae feed on transgenic maize diet, another considerable part survives and pupates. Rewritten in the new version
were collected from maize or alfalfa crops in Lleida Changed, line 106
16:8 light: dark (h) photoperiod Corrected
relative humidity (>60%) Corrected
the pupae were placed in the adult-emerging conditions at 25ºC
Rephrase this “(cylindrical methacrylate tubes of 25 cm Ø and 40 cm high, closed at the base with an expanded polystyrene lid and at the top with a white gauze) with a drinker with honey solution and a cotton ball for egg laying.” All M&M have been rewritten
80⁰C until use. -80 º C Corrected
diet made with non-Bt leaves (non-Bt), M&M have been rewritten
rewrite in scientific way (5.5 cm ø x 3cm high) and use X from symbols not from alphabets Done
correct the space between degree and Celsius throughout the text of manuscript (e.g., 25 º C) Done
Better to use symbolic multiplication instead of others……for (6 cages* 6 females/cage) and 30 males (6 cages* 5 males/cage) for each condition. & ANOVA was used (the factors were, diet * sex) Maintain space with * for two-way (parents*diet) ANOVA on development duration Done

Maintain space (Bt * β-carotene). Done
Space between fig and number (e.g., Fig1 = Fig 1) Done

Results
Percent mortality of the Helicoverpa Corrected
Discussion
Please update with latest references We have used the latest references we have found

Overall, please carefully crosscheck the reference mentioned in the text and list.

6. PLOS authors have the option to publish the peer review history of their article ([what does this mean?](https://journals.plos.org/plosone/s/editorial-and-peer-review-process#loc-peer-review-history)). If published, this will include your full peer review and any attached files.


If you choose “no”, your identity will remain anonymous but your review may still be made public.


**Do you want your identity to be public for this peer review?** For information about this choice, including consent withdrawal, please see our [Privacy Policy](https://www.plos.org/privacy-policy).

Reviewer #1: No

Reviewer #2: **Yes:** Abid Ali
